# Immune paralysis in trauma patients; implications for prehospital intervention

**DOI:** 10.1186/cc11947

**Published:** 2013-03-19

**Authors:** M Kox, K Timmermans, M Vaneker, GJ Scheffer, P Pickkers

**Affiliations:** 1 Radboud University Nijmegen Medical Center, Nijmegen, the Netherlands

## Introduction

Multi-trauma is one of the major indications for intensive care admission. Recovery is frequently complicated by post-injury immunological complications, caused by a dysfunctional immune system; for example, sepsis and multiple organ failure. In order to treat or prevent this immune paralysis, knowledge on the time course of immune paralysis *in vivo *and the pathophysiological mechanisms of immune paralysis is essential. The aim of this study is to determine factors that could predict and/or induce immunological complications in these patients to ultimately find a suitable target and timeframe for intervention.

## Methods

Blood was drawn from adult multi-trauma patients (*n *= 94) admitted to the emergency room (ER) of the Radboud University Nijmegen Medical Center. Blood was drawn at the trauma scene by the helicopter emergency medical services (HEMS), at arrival in the ER and at days 1, 3, 5, 7, 10 and 14 after trauma. Plasma concentrations of TNFα, IL-6, IL-10, IFNγ, IL-8 and MCP-1 were determined by Luminex. *Ex vivo *24-hour whole blood stimulations with LPS or pam3cys were performed and produced TNFα, IL-6 and IL-10 was measured using ELISA to determine the level of immune paralysis. Clinical data - for example, Injury Severity Scores, trauma mechanism, medication and survival - were collected from electronic patient files.

## Results

The plasma IL-10 concentration at ER was 16.5-fold increased in comparison with time-point HEMS (*P <*0.01). Similar but less pronounced effects were found for IL-8 and MCP-1. A significant correlation (*P *= 0.03, *R *= 0.53) was found between injury severity scores and IL-10 plasma concentration at time-point ER. Time-courses of *ex vivo *produced cytokines suggest that LPS-induced IL-6 and TNFα production is already decreased in the first few hours after trauma and recovering from day 5. *Ex vivo *IL-10 production shows an inverse pattern.

## Conclusion

Immune paralysis can be established within hours after trauma. Production of anti-inflammatory IL-10 in the prehospital phase could play a crucial role in the pathogenesis. Patients with a higher injury severity score are more prone to produce excessive IL-10 in this phase. Immune stimulatory strategies applied by the HEMS or early after hospital admission could form a potential future approach to prevent immune paralysis in multitrauma patients in the intensive care ward.

